# Quality of Korean and English online health information on pediatric flatfoot retrieved from NAVER and Google using the EQIP tool: a cross-sectional study

**DOI:** 10.12701/jyms.2026.43.50

**Published:** 2026-07-30

**Authors:** Yoon Ki Kim, Dong-Hwan Kim, Changsin Lee, Seung Hoo Lee

**Affiliations:** 1 Department of Orthopedic Surgery, Regional Rheumatoid and Degenerative Arthritis Center, Chungnam National University Hospital, Chungnam National University School of Medicine, Daejeon, Korea; 2 Department of Orthopedic Surgery, Chungnam National University Sejong Hospital, Chungnam National University College of Medicine, Sejong, Korea

**Keywords:** Flatfoot, Foot, Internet, Patient education, Pediatrics

## Abstract

**Background:**

Parents increasingly rely on the internet for health-related information about their children; however, the quality of such information remains inconsistent. Pediatric flatfoot is a common parental concern in pediatric orthopedics. This study compared the quality of NAVER-retrieved Korean-language websites and Google-retrieved English-language websites on pediatric flatfoot using the Ensuring Quality Information for Patients (EQIP) tool.

**Methods:**

Websites related to pediatric flatfoot were searched using NAVER for Korean-language information and Google for English-language information. After applying the predefined exclusion criteria, 50 websites from each group were evaluated using the 36-item EQIP tool, which assesses content, identification, and structure. Total and domain scores were compared between the two groups and according to institution type.

**Results:**

Google-retrieved English-language websites had significantly higher total EQIP scores than NAVER-retrieved Korean-language websites (55.50±10.17 vs. 44.88±11.83, *p*<0.001), with higher content and structure scores but similar identification scores. Among NAVER-retrieved Korean-language websites, public institution websites had higher total EQIP scores than private websites (55.38±8.69 vs. 39.95±9.74, *p*<0.001), mainly owing to higher content scores. Among the top 10 search results in each group, Google-retrieved English-language websites had higher EQIP scores and included more public institution websites than NAVER-retrieved Korean-language websites.

**Conclusion:**

In the selected search environment, Google-retrieved English-language websites had higher EQIP scores than NAVER-retrieved Korean-language websites. Among the NAVER-retrieved Korean-language websites, public institution websites provided higher-quality information than private websites, but their visibility in the NAVER search results was limited. Improving the quality and visibility of reliable Korean online resources may support informed parental decision-making.

## Introduction

In modern healthcare, patients and caregivers are increasingly turning to the internet as the primary source of health information. Although the digital revolution has greatly improved access to medical knowledge, it has also introduced significant challenges. As the internet is vast and largely unregulated, the quality, accuracy, and reliability of information are highly variable [1]. Although this accessibility offers clear benefits, the lack of systematic quality control poses significant risks [2]. Such heterogeneity of information can lead to negative consequences, including patients making flawed medical judgments and experiencing unnecessary anxiety. In Korea, seeking internet-based health information is highly prevalent. According to the 2024 Survey on internet usage conducted by the Ministry of Science and ICT, 59.3% of Korean internet users searched for health- or medical-related information online [3]. This issue is particularly relevant in pediatric orthopedics, where parents often seek health information on behalf of their children. Among pediatric orthopedic conditions, foot deformities have been reported as one of the primary concerns of parents [4].

Pediatric flatfoot is one of the most common foot deformities and is characterized by a decreased or absent medial longitudinal arch, resulting in a flattened plantar surface. The development of the medial longitudinal arch of the foot progresses over several years and exhibits a wide range of normal variations [5]. It was found that approximately 54% of children aged around 3 years and 24% of children aged around 6 years have flat feet [6,7]. By 10 years of age, flatfoot occurs in approximately 4% of children, with fewer than 1% of them requiring treatment [8-10]. Despite its generally favorable natural course, pediatric flatfoot remains a frequent source of parental concern.

Many parents seek medical care after learning about flatfoot through online resources. A systematic review indicated that 14% to 52% of patients experienced high levels of health-related anxiety [11]. Many parents frequently use the internet to understand their children’s symptoms and decide whether professional medical care is necessary. Consequently, online health information can substantially influence pediatric health outcomes because parents often make medical decisions for their children based on the information encountered online [12,13].

According to data from the Korean Health Insurance Review and Assessment Service, the number of patients seeking medical care for flatfoot has increased steadily from 9,121 in 2010 to 23,249 in 2019. In 2019, children and adolescents accounted for 76% of all cases, and this number has continued to increase annually. This trend suggests increasing healthcare utilization in pediatric patients with flatfoot.

Therefore, we evaluated the quality of the online information on pediatric flatfoot. This study aims to identify, evaluate, and compare the quality of online health information provided by NAVER-retrieved Korean-language websites and Google-retrieved English-language websites. This comparison was intended to reflect the selected search environments rather than to isolate the independent effects of language.

## Methods

**Ethics statement:** This study was approved by the Institutional Review Board (IRB) of Chungnam National University Sejong Hospital (IRB No: NON2024-008). Informed consent was not required as this study did not involve human participants or identifiable personal information.

### 1. Website selection

In this study, search frequency data related to health and medical topics were obtained from the *Internet Trend* website for the period between January 1, 2024 and October 1, 2024. During this period, health- and medical-related keywords accounted for 40.51% of all search queries on domestic websites. According to the same source, NAVER was the most frequently used search engine among Korean internet users (64.09%), followed by Google (26.72%). Based on this distribution, NAVER and Google, the two most widely used search engines, were selected for the data collection.

Following the methods used in previous studies, we initially screened the first 100 unique websites retrieved from each search engine after combining the results of the three search terms because most individuals seek information within this range [14,15]. The English searches on Google were conducted using the keywords “flatfoot,” “pediatric flatfoot,” and “flatfoot treatment,” whereas the Korean searches on NAVER were conducted using the corresponding Korean keywords “평발,” “소아 평발,” and “평발 치료,” respectively. Each keyword was searched separately using the corresponding search engine and the results from the three keywords were combined separately for each search engine/language group. Duplicate websites that appeared with different keywords were removed before applying the exclusion criteria. The Korean terms were selected as direct equivalents of the English search terms and are commonly used lay search terms in Korea.

### 2. Inclusion and exclusion criteria

Websites were eligible for inclusion if they (1) were retrieved within the predefined search range; (2) provided Korean- or English-language information related to pediatric flatfoot, flatfoot, or flatfoot treatment; (3) were accessible to the general public without payment, subscription, or institutional login; and (4) contained patient- or parent-directed health information.

Websites were excluded if they (1) were duplicate uniform resource locators appearing across multiple search terms; (2) consisted solely of video content without sufficient accompanying text; (3) were simple medical product sales or advertising pages without substantive educational content; (4) were professional or academic websites primarily providing journal articles, academic papers, or specialist-directed content; (5) were sponsored links or paid advertisements; (6) were inaccessible or required payment, subscription, or institutional login to access the relevant content; or (7) did not provide health information relevant to pediatric flatfoot. Non-healthcare institutional websites, news portals, and personal blogs were not excluded based solely on the website type if they provided patient- or parent-directed information on pediatric flatfoot and met the inclusion criteria.

After removing duplicates and applying the eligibility criteria, eligible websites were reviewed in search-result order, and the top 50 eligible websites from each search engine were included for evaluation (Fig. 1). During the eligibility assessment, 27 websites were excluded from the Korean searches on NAVER and 18 from the English searches on Google. The NAVER-retrieved Korean-language websites and Google-retrieved English-language websites included in this study are presented in Supplementary Table 1.

To minimize bias and the influence of personalized search results, all searches were conducted on the same date, October 1, 2024, in private browsing mode, without logging into a personal account. The browser cookies, caches, and browsing histories were cleared prior to each search. The searches were performed using the same computer, browser, and network environment within the same geographic region, and website lists were generated directly from the search results. The database construction, analysis, and eligibility assessment of the websites were performed between October 2024 and January 2025. Two reviewers independently evaluated the included websites. Interobserver reliability was calculated using initial independent Ensuring Quality Information for Patients (EQIP) scores. To assess intraobserver reliability, one reviewer repeated the EQIP assessment after 3 weeks. Any disagreements were resolved through consensus discussion for the final analysis.

### 3. Website assessments

We evaluated the selected websites using the expanded EQIP tool, originally introduced by Moult et al. [16], to assess the clarity and completeness of patient-directed health information. Charvet-Berard et al. [17] later refined and extended this instrument to a 36-item scale encompassing the domains of content, structure, and identification, thus enabling a more comprehensive evaluation of online medical educational resources. The EQIP tool has since been widely utilized across diverse medical fields as a validated, reproducible, and comprehensive framework for evaluating the quality of patient-directed online information [18,19].

The content domain assesses whether sufficient medical information is incorporated into the resource. Each item was scored as either “yes,” “no,” or “not applicable (NA).” The number of yes responses was divided by the total number of items and multiplied by 100 to generate a score. Items marked NA were excluded from the total number of items. Scores were calculated for total, content, identification, and structure domains.

According to the nature of the website, professional societies, government agencies, and university hospitals were classified as public institutions, whereas all others were categorized as private institutions (Fig. 2). Public institutions were defined as noncommercial or publicly accountable organizations, including government agencies, professional societies, and university hospitals. Private institutions included private hospitals, private clinics, commercial healthcare platforms, and other privately operated websites. For websites with mixed institutional affiliations, the classification was based on the primary operating body of the website rather than academic affiliation alone; therefore, university hospitals were classified as public institutions, whereas privately operated hospitals or clinics with academic affiliations were classified as private institutions. The institution type was independently classified by two reviewers, and any disagreements were resolved by a consensus discussion.

### 4. Statistical analyses

Normality of continuous variables was assessed using the Shapiro-Wilk test, and equality of variance was assessed using the Levene test. Continuous EQIP scores were compared using Student *t*-tests when the normality and equal variance assumptions were satisfied. Welch *t*-tests were used when the normality assumption was satisfied, but the equal variance assumption was not. The Mann-Whitney U test was used when the normality assumption was not satisfied or when the sample size was small, including the top 10 website analysis. Categorical comparisons were performed using the chi-square test when the expected frequencies were adequate, and the Fisher exact test when the expected frequencies were small. Bonferroni correction was applied only for multiple item-level analyses within the content domain of the EQIP tool, with statistical significance defined as *p*<0.003. The intraclass correlation coefficients (ICCs) for intraobserver and interobserver reliabilities were calculated using a two-way mixed effects model for absolute agreement. For all other analyses, significance was set at *p*<0.05. All statistical analyses were performed using IBM SPSS ver. 28 (IBM Corp., Armonk, NY, USA).

## Results

We evaluated 50 NAVER-retrieved Korean-language websites and 50 Google-retrieved English-language websites using the EQIP tool. The intraobserver reliability for the EQIP assessment was excellent, with an ICC of 0.92 (95% confidence interval [CI], 0.86–0.95), and the interobserver reliability was also excellent, with an ICC of 0.85 (95% CI, 0.75–0.91), based on the 100 included websites. The distribution of EQIP scores across all 100 websites is shown in Fig. 3. The overall mean EQIP score for all 100 websites was 50.19±12.20. When the content domain alone was scaled to 100 points, the mean score was 46.94±16.42. The identification domain scored 40.16±14.81, and the structure domain scored 60.08±12.10.

When the two groups were compared, the Google-retrieved English-language websites demonstrated significantly higher scores across all domains except for the identification domain (Table 1, Fig. 4). The Google-retrieved English-language websites had higher total EQIP scores than the NAVER-retrieved Korean-language websites (55.50±10.17 vs. 44.88±11.83, *p*<0.001), as well as higher content scores (53.22±14.16 vs. 40.67±16.24, *p*<0.001) and structure scores (65.33±10.16 vs. 54.83±11.68, *p*<0.001), whereas identification scores did not differ significantly between the two groups (42.66±15.85 vs. 37.67±13.39, *p*=0.092).

Because users frequently rely on the first page of search results, we analyzed the top 10 websites in each search engine/language group separately. The top 10 Google-retrieved English-language websites showed significantly higher EQIP total, content, and structure scores than the top 10 NAVER-retrieved Korean-language websites (Table 1). Among these top-ranked results, six English websites were public institutions, compared to only one Korean website (*p*=0.029), whereas private institutions accounted for four English websites and nine Korean websites (Table 2).

Within-group comparisons according to institution type revealed contrasting results (Table 3). Among the Google-retrieved English-language websites, no significant differences were observed between public and private institutions in any EQIP domain: total (55.03±10.67 vs. 55.84±9.97, *p*=0.783), content (53.17±14.97 vs. 53.26±13.81, *p*=0.984), identification (42.06±17.97 vs. 43.10±14.45, *p*=0.822), and structure (64.29±10.25 vs. 66.09±10.19, *p*=0.540) (Table 3). In contrast, among the NAVER-retrieved Korean-language websites, clear differences were observed according to institution type. Public Korean websites had significantly higher total EQIP scores than private Korean websites (55.38±8.69 vs. 39.95±9.74, *p*<0.001), with the largest gap observed in the content domain (56.94±8.49 vs. 33.01±13.04, *p*<0.001). Structure scores were also higher among public institutions (60.42±11.18 vs. 52.21±11.11, *p*=0.019), whereas identification scores did not differ significantly (40.63±12.12 vs. 36.27±13.90, *p*=0.288) (Table 3).

A direct comparison between the Google-retrieved English-language and NAVER-retrieved Korean-language websites within the same institution type revealed no significant differences between the two groups among websites operated by public institutions across domains (Table 4). However, English private institutions scored significantly higher than Korean private institutions in total EQIP score (55.84±9.97 vs. 39.95±9.74, *p*<0.001), content (53.26±13.81 vs. 33.01±13.04, *p*<0.001), and structure (66.09±10.19 vs. 52.21±11.11, *p*<0.001). Identification scores approached but did not reach significance (43.10±14.45 vs. 36.27±13.90, *p*=0.061) (Table 4).

For the item-level analysis of the EQIP content domain, we evaluated all 18 content criteria among the NAVER-retrieved Korean-language websites by comparing those operated by public and private institutions. Several items showed significant differences after Bonferroni correction, with the largest association observed for whether a specific source of the content was provided (Cramer’s V=0.736). Item 13, “Explanation of precautions patients can take,” also showed a strong association with institution type (Cramer’s V=0.578), suggesting a gap in practical patient- or parent-directed guidance. Additional significant items included explanations of appropriate surgical methods if nonsurgical treatment was ineffective, qualitative benefits during treatment, and specific details on accessing medical services (Table 5).

## Discussion

To our knowledge, this is the first study to compare and analyze online information on pediatric flatfoot between NAVER-retrieved Korean-language websites and Google-retrieved English-language websites, yielding four noteworthy findings. First, Google-retrieved English-language websites showed higher overall EQIP scores than NAVER-retrieved Korean-language websites in the selected search environment (Table 1). Second, among the top 10 websites in each group, the Google-retrieved English-language websites consistently achieved higher EQIP scores than their NAVER-retrieved Korean-language counterparts, suggesting that search engine-specific retrieval environments influence the visibility of online health information (Table 1). Third, among the NAVER-retrieved Korean-language websites, those operated by public institutions provided higher-quality information than those run by private institutions (Table 3). Fourth, among the NAVER-retrieved Korean-language websites, item-level analysis revealed differences between public and private institutions in terms of specific EQIP content items, highlighting areas where improvement is required (Table 5).

The EQIP tool was selected because it provides a structured assessment of patient-directed health information across multiple dimensions, including content, identification, and structure [16]. Other instruments have also been used to evaluate online health information; however, they differ in their focus. DISCERN primarily evaluates the quality of written consumer health information regarding treatment choices, whereas JAMA (*Journal of the American Medical Association*) benchmarks emphasize accountability and credibility indicators, such as authorship, attribution, disclosure, and currency [20,21]. Health On the Net Code of Conduct (HONcode) has historically focused on ethical and transparency standards for health websites, including authority, complementarity, privacy, attribution, justifiability, transparency, financial disclosure, and advertising policy [22]. In contrast, the expanded 36-item EQIP tool allows a broader evaluation of the completeness and presentation of patient-directed information. This is particularly relevant to pediatric flatfoot because the quality of online information depends not only on whether treatment options are described but also on whether essential messages, such as the generally benign natural history of asymptomatic pediatric flatfoot and the lack of treatment need in many cases, are clearly communicated.

According to our analyses, the mean total EQIP score for the Google-retrieved English-language websites was 55.50±10.17, which was significantly higher than that for the NAVER-retrieved Korean-language websites (Table 1). The mean content score was 53.22 for the Google-retrieved English-language websites and 40.67 for the NAVER-retrieved Korean-language websites, reflecting a significant difference (Table 1). These findings suggest that the overall quality control of the NAVER-retrieved Korean-language websites for pediatric flatfoot is suboptimal. Consistent with previous research, low-quality or misleading health information can be harmful to both patients and physicians. Daraz et al. [23] conducted a systematic review and reported that the internet does not provide reliable health information to the public. Luo et al. [24] found that low-quality online health information can negatively impact physician–patient relationships. Bryan et al. [25] observed that most parents obtain information about their children’s health from social media and often do not discuss it with their child’s pediatrician.

For the NAVER-retrieved Korean-language websites, a significant difference was observed in the total EQIP scores, with public institutions scoring higher than private institutions (Table 3). These findings are consistent with evidence from a systematic review indicating that public sector services generally provide higher-quality, more standardized, and evidence-based care than private sector services [26]. However, this phenomenon was not observed for the Google-retrieved English-language websites (Table 3). One possible explanation is that search engine-specific retrieval environments differ between Google and NAVER. In the present study, many of the top-ranked NAVER search results were from private or commercial websites, whereas the Google search results more often included websites from authoritative hospitals, government agencies, and relevant academic societies (Table 2). Schultheiß et al. [27] reported that search engines can sometimes rank low-quality, commercial content higher than more reliable medical information, indicating that ranking position is not always a valid indicator of quality. Eysenbach and Köhler [28] noted that most users do not search extensively and typically review only a few results on the first search page. Although the present study did not directly evaluate search engine algorithms, the limited visibility of authoritative public institution websites in the NAVER search results may reflect differences in search result composition, advertising placement, ranking systems, search engine optimization, and institutional visibility.

In the item-level analysis of the EQIP content domain among the NAVER-retrieved Korean-language websites, the largest association with institution type was observed for whether a specific source of the content was provided (Cramer’s V=0.736). Item 13, “Explanation of precautions patients can take,” also showed a strong association with institution type (Cramer’s V=0.578), suggesting a gap in practical patient- or parent-directed guidance. Additional significant items included explanations of appropriate surgical methods if nonsurgical treatment was ineffective, qualitative benefits during treatment, and specific details on accessing medical services (Table 5). From a clinical perspective, these findings underscore the importance of providing practical guidance for parents. In pediatric flatfoot, such guidance should include clear communication that asymptomatic flexible flatfoot is usually a benign developmental condition that does not require treatment in many cases. Similarly, Goff et al. [29] identified comprehensiveness, accuracy, and overall quality as key determinants of high-quality online health information for lay audiences.

Given the substantial variability in information quality and the tendency for low-quality sources to be more prominent in certain search environments, clinicians should proactively ask parents which online resources they have consulted and address any misconceptions during consultations. Clinicians should guide parents toward websites operated by public or authoritative institutions, such as government or public health agencies, professional medical societies, university hospitals, and other noncommercial educational platforms, rather than relying solely on search rankings or advertisement-based search results. When counseling parents, clinicians should provide clear guidance on the benign natural history of asymptomatic pediatric flatfoot and the limited indications for treatment. Enhancing the visibility of accurate evidence-based resources, particularly those from public or authoritative institutions, may help reduce parental anxiety, discourage unnecessary treatment, and improve shared decision-making.

This study had some limitations. First, the effects of language and search engines could not be fully separated. The two comparison groups comprised Google-retrieved English-language and NAVER-retrieved Korean-language websites, which reflected the dominant search engines commonly used for each language. Therefore, the observed differences in EQIP scores may have been influenced not only by language-related factors, but also by search engine-specific retrieval environments, which were not directly evaluated in this study. Although the Korean search terms were selected as direct equivalents of the English terms and reflected the commonly used lay terminology in Korea, subtle semantic and cultural differences between the search terms may have influenced the types of websites retrieved. Second, although EQIP provides a structured assessment of patient-directed information, it does not directly assess clinical accuracy, readability, patient comprehension, or behavioral impact. Moreover, because published studies using the expanded 36-item EQIP tool remain limited, the observed mean total EQIP score is difficult to benchmark against robust normative data or to classify as high, moderate, or low quality; therefore, the score should be interpreted as a relative indicator of information completeness and quality. Finally, given the dynamic nature of the internet, our findings, which were based on data collected between October 2024 and January 2025, may not fully reflect the continuously evolving landscape of online health information. Changes in search algorithms, paid advertisement policies, and availability or ranking of health-related websites may affect the visibility and quality of information encountered by parents over time. Therefore, periodic reassessments, such as annual audits or reassessments following major changes in search engine policies or website ecosystems, may be valuable for monitoring the quality of online health information and maintaining the practical relevance of these findings.

In conclusion, in the selected search environment, Google-retrieved English-language websites showed higher overall EQIP scores than NAVER-retrieved Korean-language websites. This difference may reflect not only language-related factors, but also differences in search engine-specific retrieval environments. Among the NAVER-retrieved Korean-language websites, public institutions provided higher-quality information than private websites, but their visibility in the NAVER search results was limited. These findings may help parents to identify higher-quality online health information and highlight the need to improve both the quality and search visibility of reliable Korean online resources.

## Figures and Tables

**Fig. 1.**
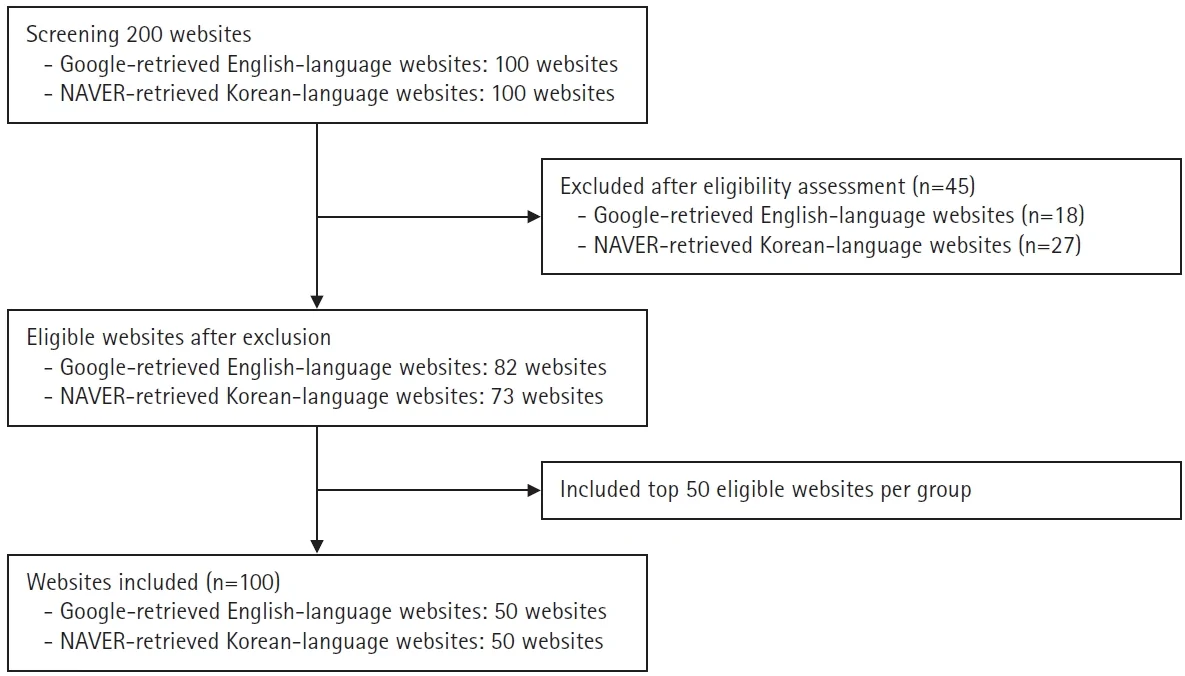
Flowchart of website screening and evaluation. The first 100 unique websites retrieved from each search engine are screened, and the top 50 eligible websites from each search engine are included for Ensuring Quality Information for Patients (EQIP) evaluation.

**Fig. 2.**
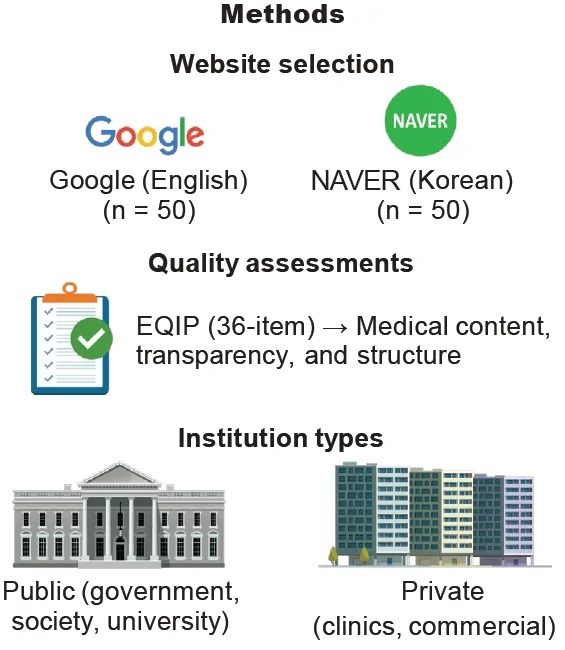
Overview of the study methods used to evaluate Google-retrieved English-language websites and NAVER-retrieved Korean-language websites providing online information on pediatric flatfoot. EQIP, Ensuring Quality Information for Patients.

**Fig. 3.**
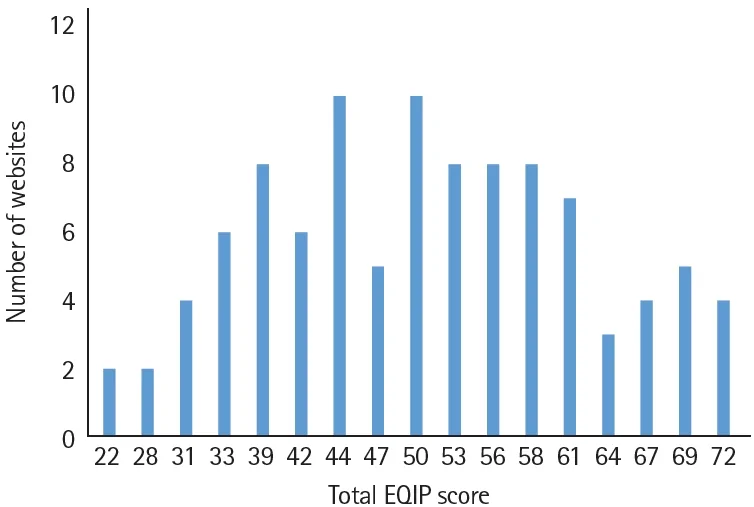
Frequency distribution of total Ensuring Quality Information for Patients (EQIP) scores. The histogram shows the number of websites according to their total EQIP scores.

**Fig. 4.**
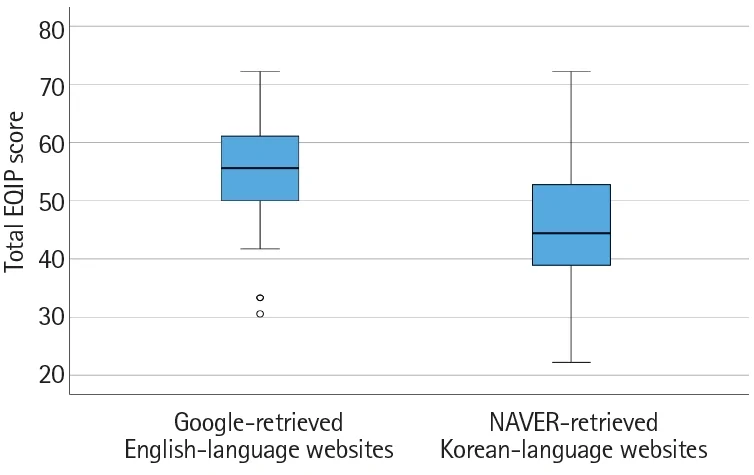
Box plot of total Ensuring Quality Information for Patients (EQIP) scores. Google-retrieved English-language websites score significantly higher than NAVER-retrieved Korean-language websites (55.50±10.17 vs. 44.88±11.83, *p*<0.001).

**Table 1.** Comparison of EQIP scores between Google-retrieved English-language websites and NAVER-retrieved Korean-language websites (all websites vs. top 10 search results appearing first in search results)

Domain	All websites			Top 10 websites		
	Google-retrieved English-language websites	NAVER-retrieved Korean-language websites	*p*-value	Google-retrieved English-language websites	NAVER-retrieved Korean-language websites	*p*-value
Total score	55.50±10.17	44.88±11.83	<0.001	59.44±9.99	45.56±9.18	0.005
Content	53.22±14.16	40.67±16.24	<0.001	58.33±13.16	43.33±11.94	0.016
Identification	42.66±15.85	37.67±13.39	0.092	46.67±17.21	36.67±15.32	0.187
Structure	65.33±10.16	54.83±11.68	<0.001	67.50±8.29	53.33±12.55	0.008

Values are presented as mean±standard deviation. EQIP, Ensuring Quality Information for Patients.

**Table 2.** Distribution of public and private institutions in the top 10 search results: Google-retrieved English-language websites vs. NAVER-retrieved Korean-language websites

Institution	Google-retrieved English-language websites	NAVER-retrieved Korean-language website	*p*-value
Public	6	1	0.029
Private	4	9	

**Table 3.** Comparison of EQIP scores between public and private institutions in Google-retrieved English-language websites and NAVER-retrieved Korean-language websites

Domain	Google-retrieved English-language websites			NAVER-retrieved Korean-language websites		
	Public institutions	Private institutions	*p*-value	Public institutions	Private institutions	*p*-value
Total score	55.03±10.67	55.84±9.97	0.783	55.38±8.69	39.95±9.74	<0.001
Content	53.17±14.97	53.26±13.81	0.984	56.94±8.49	33.01±13.04	<0.001
Identification	42.06±17.97	43.10±14.45	0.822	40.63±12.12	36.27±13.90	0.288
Structure	64.29±10.25	66.09±10.19	0.540	60.42±11.18	52.21±11.11	0.019

Values are presented as mean±standard deviation. EQIP, Ensuring Quality Information for Patients.

**Table 4.** Comparison of EQIP scores between Google-retrieved English-language websites and NAVER-retrieved Korean-language websites in public and private institutions

Domain	Public institutions			Private institutions		
	Google-retrieved English-language websites	NAVER-retrieved Korean-language website	*p*-value	Google-retrieved English-language websites	NAVER-retrieved Korean-language website	*p*-value
Total score	55.03±10.67	55.38±8.69	0.914	55.84±9.97	39.95±9.74	<0.001
Content	53.17±14.97	56.94±8.49	0.340	53.26±13.81	33.01±13.04	<0.001
Identification	42.06±17.97	40.63±12.12	0.773	43.10±14.45	36.27±13.90	0.061
Structure	64.29±10.25	60.42±11.18	0.282	66.09±10.19	52.21±11.11	<0.001

Values are presented as mean±standard deviation. EQIP, Ensuring Quality Information for Patients.

**Table 5.** Item-level comparison of EQIP content characteristics between public and private institutions within NAVER-retrieved Korean-language websites

Content (18 criteria)	Public institutions	Private institutions	*p*-value^a)^	Cramer’s V
Presents definition of the topic	16/16	32/34	0.322	0.140
Includes content related to the defined topic	16/16	32/34	0.322	0.140
Presents medical problems/treatment methods and processes	16/16	24/34	0.015	0.343
Explanation of nonsurgical treatment	16/16	24/34	0.015	0.343
Explanation of surgical treatment	10/16	8/34	0.007	0.379
Explanation of appropriate surgical methods if nonsurgical treatment is ineffective	9/16	2/34	<0.001	0.567
Explanation of qualitative benefits for patients during treatment	15/16	16/34	0.002	0.449
Explanation of quantitative benefits for patients during treatment	3/16	2/34	0.157	0.200
Qualitative side effects or risks for patients during treatment	0/16	0/34	-	-
Quantitative side effects or risks for patients during treatment	1/16	0/34	0.141	0.208
Mentions impact on quality of life during treatment intervention	11/16	10/34	0.009	0.372
Mentions coping strategies for complications	1/16	0/34	0.141	0.208
Explanation of precautions patients can take	13/16	7/34	<0.001	0.578
Mentions signs patients can detect	12/16	12/34	0.009	0.371
Mentions medical costs or insurance issues	0/16	0/34	-	-
Specific details on accessing medical services	3/16	22/34	0.002	0.429
Whether there is a specific source for the content	14/16	4/34	<0.001	0.736
Whether major issues related to the topic were covered	8/16	7/34	0.034	0.299

EQIP, Ensuring Quality Information for Patients.

a) Statistical significance was determined using Bonferroni correction, with a threshold of *p*<0.003.

## Supplementary materials

Supplementary Table 1 can be found at https://doi.org/10.12701/jyms.2026.43.50.

Supplementary Table 1. Naver-retrieved Korean-language websites and Google-retrieved English-language websites included in this study
