# Supplementary material for: Quality of Korean and English online health information on pediatric flatfoot retrieved from NAVER and Google using the EQIP tool: a cross-sectional study
Source: J Yeungnam Med Sci. 2026 Jul 30;43:50. doi: 10.12701/jyms.2026.43.50 (PMC13681076; doi:10.12701/jyms.2026.43.50)
Supplement: Supplementary file 1 [file jyms-2026-43-50-Supplementary-Table-1.pdf]

Supplementary Table 1. NAVER-retrieved Korean-language websites and Google-retrieved English-language websites included in this study

No	NAVER-retrieved Korean-language websites (n = 50)	Google-retrieved English-language websites (n = 50)
1	http://healthyfriend.co.kr/footcenter/foot_15	https://pediatricfootankle.com/foot-conditions/pediatric-flat-feet/
2	https://seoul1hospital.com/sub/s3/s2/s1/s1.php	https://www.chop.edu/conditions-diseases/flat-feet-in-children
3	http://i-leg.kr/bbs/content.php?co_id = d_11	https://kidshealth.org/en/parents/flat-feet.html
4	http://www.snuh.org/health/nMedInfo/nView.do?category = DIS&medid = AA000197	https://www.rch.org.au/uploadedFiles/Main/Content/rheumatology/Flat_feet_in_children.pdf
5	https://blog.naver.com/ysoksok7807/223513346852	https://www.ouh.nhs.uk/paediatricorthopaedics/information/conditions/flat-foot.aspx
6	https://blog.naver.com/hdream613/223582861399	https://orthoinfo.aaos.org/en/diseases--conditions/flexible-flatfoot-in-children
7	https://blog.naver.com/jhmed_no1/223399780305	https://upmc.it/en/blog/pediatrics/what-causes-flat-feet-in-children
8	https://blog.naver.com/soroco/223619827944	https://www2.hse.ie/conditions/flat-feet-in-children/
9	https://blog.naver.com/hchpublic/223289211584	https://www.foothealthfacts.org/conditions/pediatric-flatfoot
10	https://blog.naver.com/kmsungae0112/223597327986	https://ankleandfootcenterstn.com/pediatric-flat-foot/
11	https://blog.naver.com/allbareunns/223629095425	https://rhc.nhsggc.org.uk/home/about-your-childs-health-information-resources/patient-information-leaflets/physiotherapy/apcp/flat-feet-in-children-information-for-parents-and-carers/
12	https://post.naver.com/viewer/postView.naver?volumeNo = 39351774&memberNo = 38488371&vType = VERTICAL	https://myhealth.alberta.ca/Health/aftercareinformation/pages/conditions.aspx?hwid = tf7241
13	https://terms.naver.com/entry.naver?docId = 6629362&cid = 64672&categoryId = 64672	https://www.schn.health.nsw.gov.au/flat-feet-factsheet
14	https://www.amc.seoul.kr/asan/depts/child/K/bbsDetail.do?menuId = 4342&contentId = 259898	https://www.nuh.nhs.uk/flatfoot-in-children/
15	https://blog.naver.com/noxhyang/223348645520	https://www.healthychildren.org/English/health-issues/conditions/orthopedic/Pages/Flat-Feet-Fallen-Arches.aspx
16	https://www.chungnamilbo.co.kr/news/articleView.html?idxno = 796306	https://www.mountelizabeth.com.sg/health-plus/article/flat-feet-common-children
17	http://www.kyeongin.com/main/view.php?key = 20241016010001366	https://www.afacutah.com/blog/flat-feet-in-kids-to-treat-or-not-to-treat
18	https://mdtoday.co.kr/news/view/1065574050385785	https://www.pregnancybirthbaby.org.au/flat-feet-in-children
19	https://www.hidoc.co.kr/healthstory/news/C0000865819	https://thefootpractice.com/childrens-flat-feet/
20	https://www.dubalo.com/view/sub0501-06.php	https://www1.racgp.org.au/ajgp/2020/may/paediatric-pes-planus
21	https://blog.naver.com/sakuraba23/223115223345	https://www.orthobullets.com/pediatrics/4069/flexible-pes-planovalgus-flexible-flatfoot
22	https://blog.naver.com/dlgml121612/223204437943	https://apcp.csp.org.uk/content/flat-feet-children
23	https://health.kdca.go.kr/healthinfo/biz/health/gnrlzHealthInfo/gnrlzHealthInfo/gnrlzHealthInfoView.do?cntnts_sn = 5840	https://www.rch.org.au/ortho/for_health_professionals/Flat_feet/
24	https://www.sportsworldi.com/newsView/20231027503362	https://www.eastcoastpodiatry.sg/paediatric-flat-feet/
25	http://www.dalrija.com/sub/r70/s70101030.php	https://cliftonfootandankle.com/edu_patient/pediatric-flat-feet/
26	https://blog.naver.com/medifoot/223286952589	https://www.fixingfeet.com/blog/treating-flat-feet-in-children.cfm
27	https://www.jeonmin.co.kr/news/articleView.html?idxno = 344451	https://www.columbiadoctors.org/treatments-conditions/flat-feet-pediatric
28	https://sports.donga.com/society/article/all/20230717/120271806/2	https://theconversation.com/what-are-flat-feet-in-children-and-are-they-something-to-worry-about-60365
29	https://news.heraldcorp.com/view.php?ud = 20181122000362	https://orthotic.ca/condition-guides/pediatric-flat-foot/
30	https://happysoyoung.tistory.com/entry/%EC%95%84%EA%B8%B0-%ED%8F%89%EB%B0%9C-%EC%9B%90%EC%9D%B8%EA%B3%BC-%EA%B4%80%EB%A6%AC%EB%B0%A9%EB%B2%95-%EC%95%8C%EA%B3%A0-%EB%8C%80%EC%B2%98%ED%95%98%EA%B8%B0	https://www.childrenshospital.org/conditions/flexible-flatfoot
31	https://m.post.naver.com/viewer/postView.nhn?volumeNo = 36515402&memberNo = 1544782	https://www.samitivejhospitals.com/article/detail/the-truth-about-flat-feet-in-children-what-parents-need-to-know

(Continued to the next page)

Supplementary Table 1. Continued

No	NAVER-retrieved Korean-language websites (n = 50)	Google-retrieved English-language websites (n = 50)
32	https://blogdo.tistory.com/entry/%EC%9A%B0%EB%A6%AC-%EC%95%84%EC%9D%B4-%ED%8F%89%EB%B0%9C%EC%9D%B8%EC%A7%80-%EC%B2%B4%ED%81%AC%ED%95%98%EA%B8%B0-%EC%86%8C%EC%95%84%ED%8F%89%EB%B0%9C-%EA%B5%90%EC%A0%95%ED%95%98%EA%B8%B0	https://my.clevelandclinic.org/health/diseases/17005-flat-feet
33	https://www.keyboneos.co.kr/44	https://www.milestonepedstherapy.com/blog/2019/1/15/pediatric-flat-foot
34	https://orthopedics210.com/110	https://www.foundationpodiatry.com.au/conditions/children-s-conditions/flat-feet-in-children/
35	https://www.ibabynews.com/news/articleView.html?idxno = 71936	https://www.yourfamilyfootcare.com/library/pediatric-flat-feet.cfm
36	http://www.thegolftimes.co.kr/news/articleView.html?idxno = 58473	https://summithealthcare.net/blog/podiatry/treating-pediatric-flat-feet/
37	https://www.donga.com/news/article/all/20210519/107018120/1	https://www.oxfordhealth.nhs.uk/podiatry/wp-content/uploads/2013/12/Flatfeet-in-children.pdf
38	https://seoulhyun.kr/view/sub0507.php	https://certifiedfoot.com/services/pediatric-flat-feet/
39	https://sonata2002.tistory.com/entry/%EA%B5%90%EC%A0%95%ED%99%95%EB%A5%A0%EC%9D%B4-%EB%A7%A4%EC%9A%B0-%EB%86%92%EC%9D%80-%E2%80%98%EC%86%8C%EC%95%84%ED%8F%89%EB%B0%9C%E2%80%99	https://www.straitspodiatry.com/conditions/paediatric-flat-feet/
40	https://www.snuseoul.kr/page/sub0202-5.php	https://www.umms.org/ummc/health-services/orthopedics/services/pediatric/flat-feet
41	https://www.rapportian.com/news/articleView.html?idxno = 150689	https://orthokids.org/conditions/flexible-flat-feet/
42	http://www.samsunghortho.com/bbs/content.php?co_id = page20	https://p-ortho.com/paediatric-orthopaedic-services/kids-flat-foot/
43	https://www.medical-tribune.co.kr/news/articleView.html?idxno = 106517	https://www.luriechildrens.org/en/specialties-conditions/pes-planus/
44	http://www.gnsooclinic.com/young/young04.asp	https://blog.dinopt.com/flat-feet-you-say/
45	http://anapaborn.com/theme/anapaborn/sub/child_6030.php	https://www.shrinerschildrens.org/en/pediatric-care/pes-planus-pes-planovalgus
46	http://www.doctorgood.com/doc/foot6.html	https://newyorkfootcareservices.com/pediatric-flat-feet/
47	https://field2580.tistory.com/1148	https://www.nextstepfoot.com/flat-footed-kids-dont-worry/
48	https://daonsamsung.com/%EC%86%8C%EC%95%84%EC%A0%95%ED%98%95%EC%99%B8%EA%B3%BC/	https://www.prolaborthotics.com/resource/pediatric-flatfoot-pathology/
49	https://www.dabonclinic.com/foot/toein/index.jsp	https://www.clarkpodiatry.com/flat-feet-in-children/
50	https://footsallyeonsinnae.tistory.com/entry/%ED%8F%89%EB%B0%9C%EC%9D%B4%EB%9E%80-%EB%AC%B4%EC%97%87%EC%9D%BC%EA%B9%8C%EC%9A%94-%EB%B0%9C%EA%B5%AC%EC%A1%B0	https://www.thefeetpeople.com.au/symptoms-we-treat/flat-feet-children/

https://doi.org/10.12701/jyms.2026.43.50
